# Supplementary material for: Deletion of wfs1 Impairs Oligodendrocyte Precursor Cells Dorsal Distribution and Myelination Through the wfs1-hmgcs1 Axis in Zebrafish
Source: Biology (Basel). 2026 Aug 16;15(16):1402. doi: 10.3390/biology15161402 (PMC13510151; doi:10.3390/biology15161402)
Supplement: Supplementary file 1 [file biology-15-01402-s001.zip › File S1-Transcriptome sequencing data and differentially expressed genes (DEGs) in wfs1-- zebrafish at 5 dpf/1.GO/wfs1KOvsWT/UP/wfs1KOvsWT.up_GObar_bp.pdf]

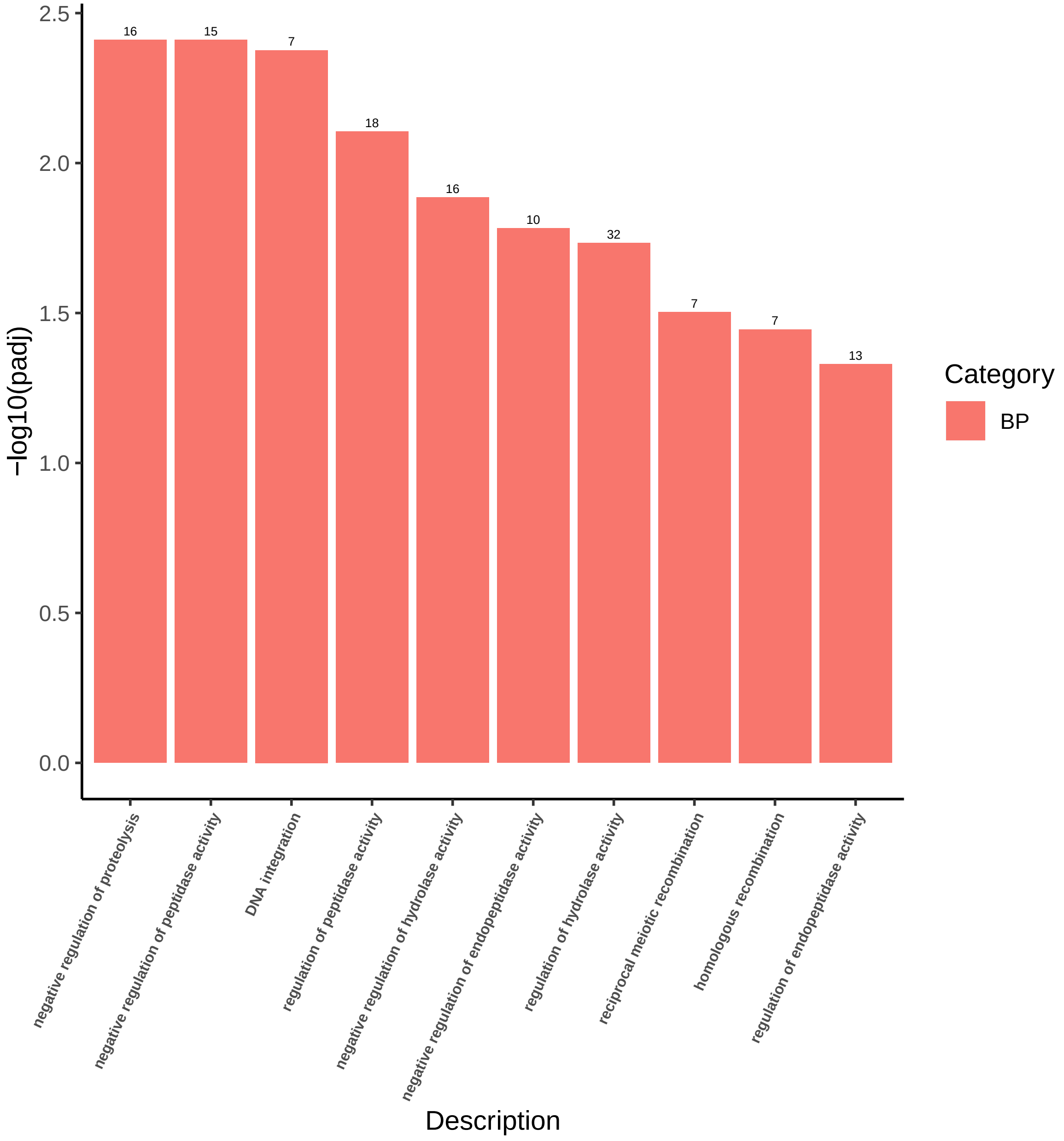

-log10(padj)
2.5
2.0
1.5
1.0
0.5
0.0
16
15
7
18
16
10
32
7
7
13
negative regulation of proteolysis
negative regulation of peptidase activity
DNA integration
regulation of peptidase activity
negative regulation of hydrolase activity
negative regulation of endopeptidase activity
regulation of hydrolase activity
reciprocal meiotic recombination
homologous recombination
regulation of endopeptidase activity
Description
Category
BP
